# Unusual Presentation of Hepatocellular Carcinoma and Rare Metastasis to the Masticatory Space

**DOI:** 10.7759/cureus.5802

**Published:** 2019-09-30

**Authors:** Urban Čizmarević, Nina Hanžič, Beno Polanec, Mitja Rupreht

**Affiliations:** 1 Radiology, University Medical Centre Maribor, Maribor, SVN

**Keywords:** hepatocellular carcinoma, extrahepatic metastasis, liver metastasis, masticatory space, unusual presentation, extrahepatic manifestations

## Abstract

An incidental liver mass was discovered in a 65-year-old male during a routine ultrasound (US) check-up of his hiatal hernia. The mass, which showed no malignant characteristics, was interpreted as a focal nodular hyperplasia (FNH). Due to normal blood tests and tumor marker levels, as well as the patient's asymptomatic presentation, only regular monitoring was performed. At a check-up 18 months later, CT examination indicated hepatocellular carcinoma (HCC). Surgery was no longer possible due to diffuse liver involvement. Transarterial chemoembolization (TACE) and chemotherapy were started. A possible metastasis to the right adrenal gland was detected. The patient started to experience headaches, vertigo, paresthesia, and pain of the right jaw. A CT scan of the head showed a mass in the right masticatory space. A CT-guided biopsy confirmed a HCC metastasis.

## Introduction

Hepatocellular carcinoma (HCC) is the most common primary tumor of the liver. It has a very poor five-year survival rate, second only to pancreatic cancer. More than 782,000 people worldwide died from liver cancer in 2018, with the majority due to HCC [1-2]. In most cases, it arises in the setting of chronic liver disease, usually associated with alcohol addiction or hepatitis B or C infections. Rarely malignant transformation from hepatic adenoma occurs [3]. Due to the large functional reserve of the liver, the diagnosis is often made late, and only around one-fifth of the patients are candidates for surgery [4]. At the time of diagnosis, extrahepatic metastases are found in around 15% of the patients with the most common sites being the lungs, distant lymph nodes, and bones [5].

## Case presentation

**Figure 1:**
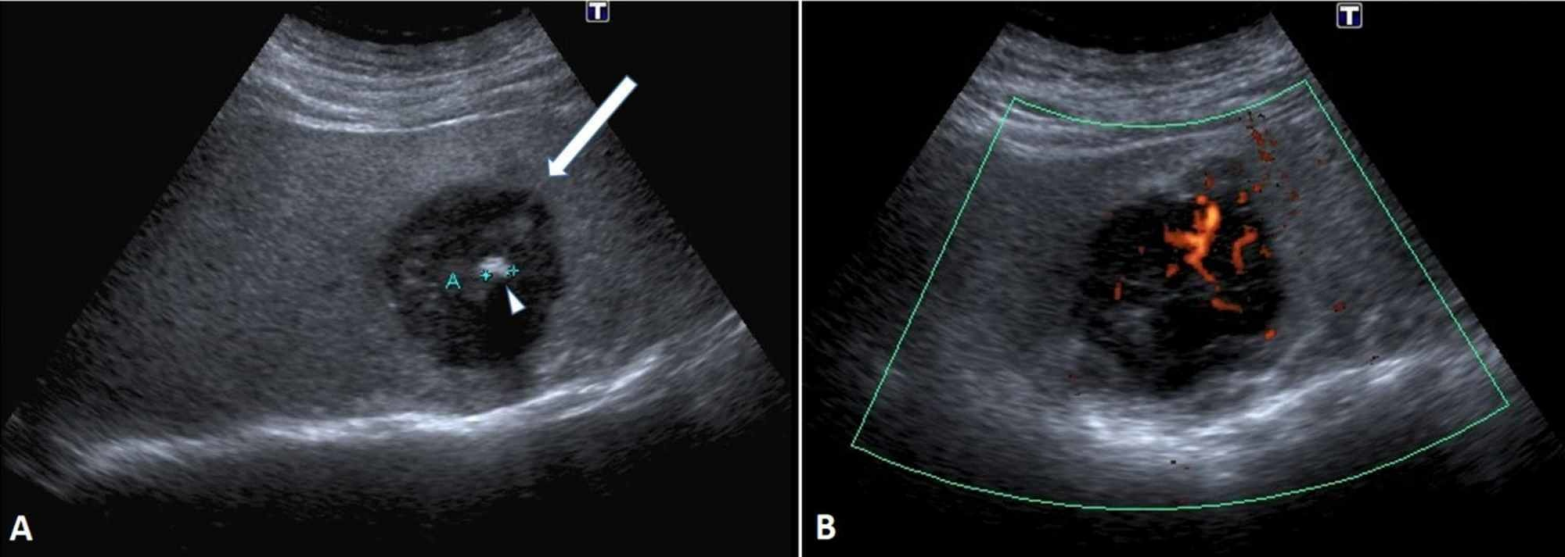
Liver mass. (A) US exam shows diffuse hyperechogenic (fatty) liver parenchyma and moderately hypoechogenic mass (arrow) with central hyperechogenic core (arrowhead). At the power Doppler (B) a central artery with spoke-wheel (centrifugal) flow is demonstrated. US, ultrasound

A 65-year-old male had a check-up for a large hiatal hernia. He had diabetes and was suffering from benign prostatic hyperplasia (BPH). An abdominal ultrasound (US) examination revealed an incidental hypoechogenic mass with irregular borders in the sixth segment of the liver, measuring 5.5 cm x 5 cm (Figure 1A). The lesion had a central hyperechogenic core of 8 mm. No capsule or compression of surrounding liver tissue was visible. Both Doppler US and contrast-enhanced US (CEUS) examinations showed arterial supply from the hepatic artery and spoke-wheel or centrifugal flow from the central artery, characteristic for focal nodular hyperplasia (FNH) (Figure 1B) [6]. Another smaller (0.8 cm x 1.5 cm) similar lesion was noticed in the seventh liver segment. The liver parenchyma appeared steatotic throughout.

**Figure 2:**
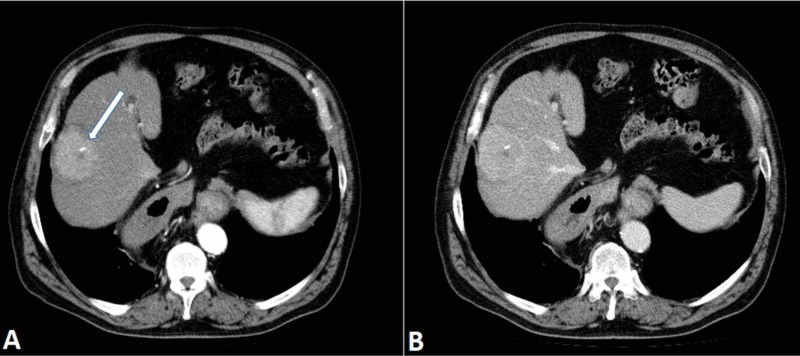
Liver mass. Axial CT scan in the arterial phase (A) demonstrates a mildly hyperdense mass in the sixth liver segment with a central calcification (arrow). The mass shows early arterial enhancement, but a typically fast wash-out characteristic for HCC is not seen (B). HCC, hepatocellular carcinoma

A CT scan performed the following day confirmed the presence of a nonhomogenous change in the sixth segment of the liver (Figure 2). Tumor marker levels were not increased.

**Figure 3:**
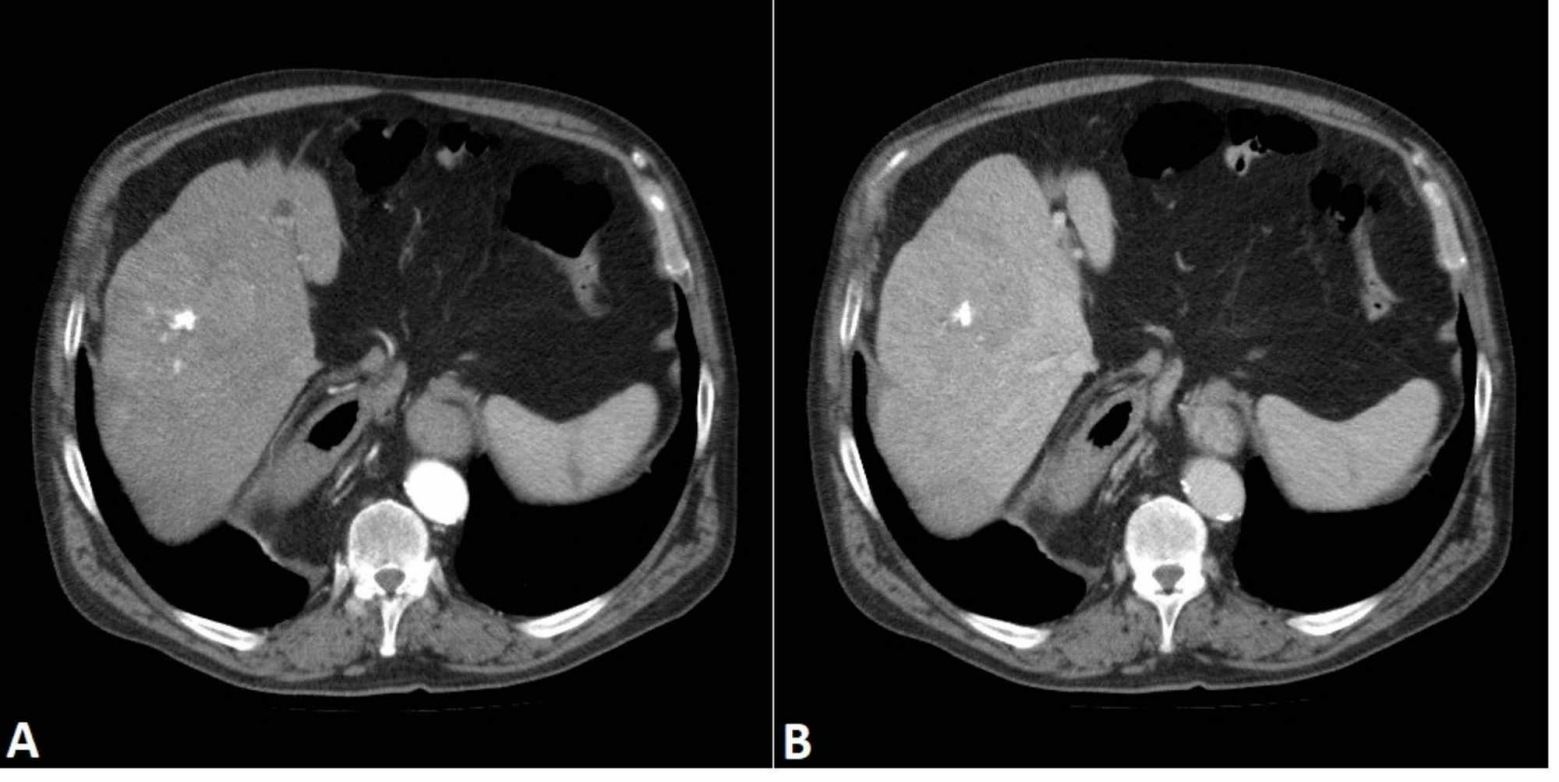
Liver mass. Follow-up CT examination shows enlargement of the main lesion (measuring 8 cm x 10 cm). The lesion has a calcified center with hyperdense septa spreading outwards. It demonstrates early enhancement in the arterial phase (A) with signs of quick wash-out in the portal phase (B).

The patient's clinical and laboratory findings were unremarkable. He used to be a smoker but had stopped more than 20 years ago. He denied excessive use of alcohol. The blood tests for a hepatitis infection were negative. Owing to the typical findings of the FNH, the lesion was considered benign, and only regular check-ups were performed every six months. The lesion did not show any notable changes at the first two check-ups, but 18 months later an enlargement was detected by US and another CT scan was performed. It revealed a sizable increase of the main lesion, now measuring 8 cm x 10 cm (Figure 3). Before the contrast application, the lesion was hyperdense compared to the surrounding (not shown). The lesion had a calcified center with hyperdense septa spreading outwards. It demonstrated early enhancement in the arterial phase (Figure 3A) with signs of quick wash-out in the portal phase (Figure 3B). In addition, several hypodense foci were visible in the portal phase, indicating necrotic areas. The smaller lesion had also enlarged in size, now measuring 2.2 cm x 2.5 cm. More satellite lesions measuring up to 1 cm were also seen throughout the liver parenchyma. A needle biopsy was considered but was decided against due to a possibility of seeding or bleeding complications and an already very convincing imaging presentation. The diagnosis of HCC was made. Due to diffuse involvement of the liver, surgery was not indicated; transarterial chemoembolization (TACE) and chemotherapy with sorafenib were initiated instead.

**Figure 4:**
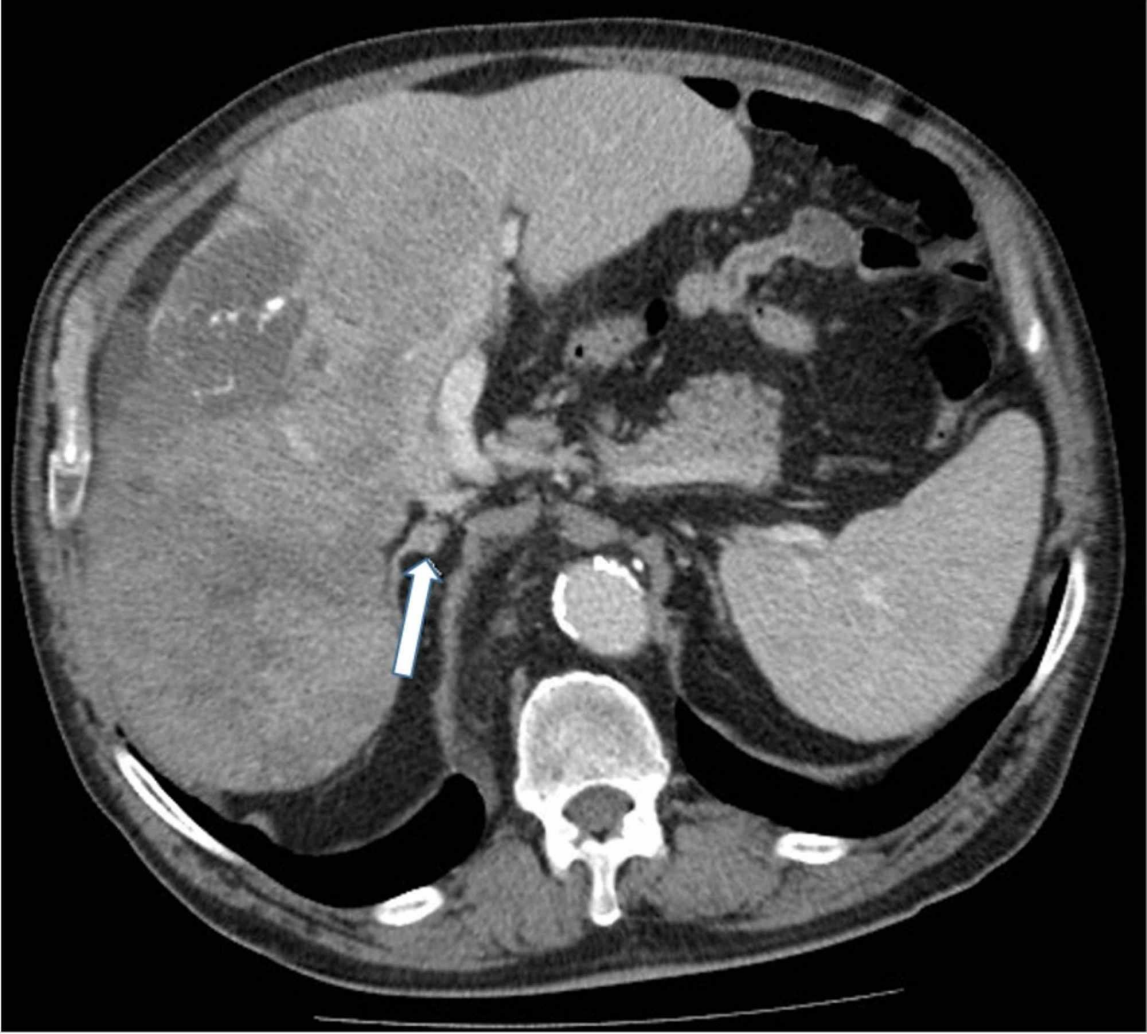
Mass in the right adrenal gland. A small mass (arrow) in the right adrenal gland was visible in the axial CT.

**Figure 5:**
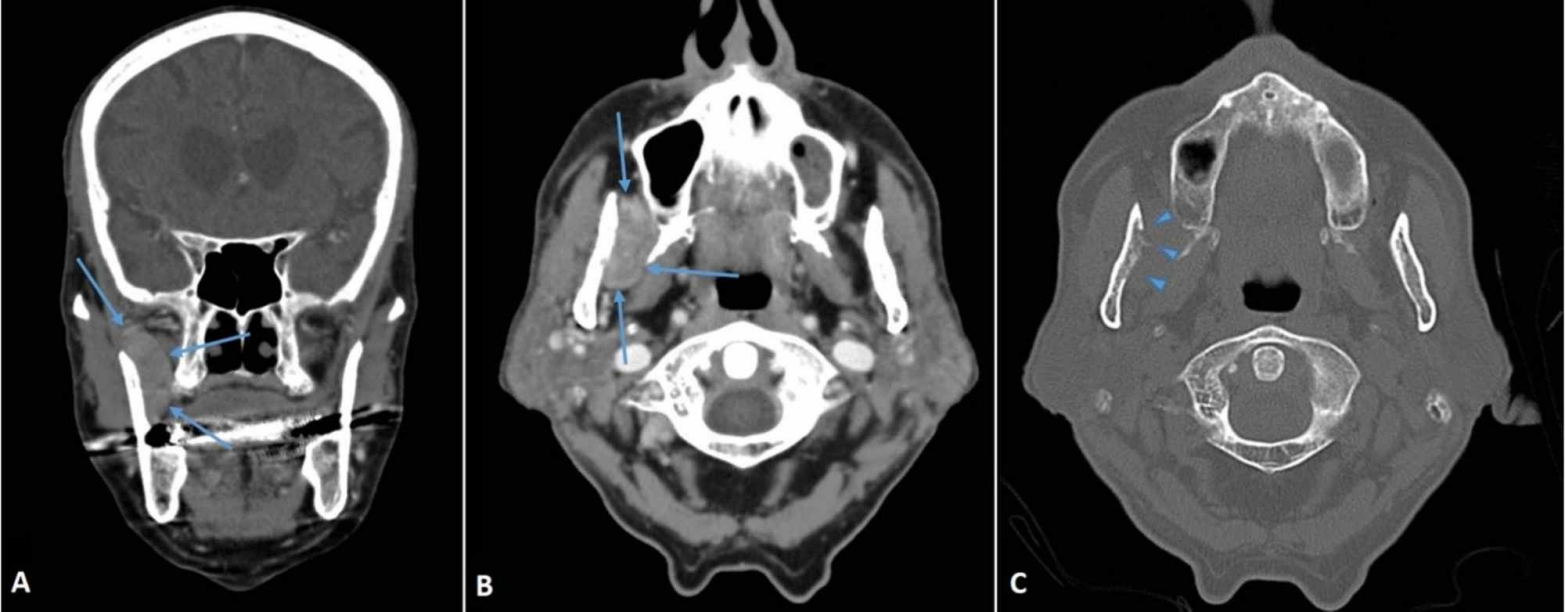
Masticatory space metastasis. (A, B) CT scan of the neck showing a mass in the right masticatory space; lateral to the lateral pterygoid muscle, and medial to the ramus of the mandible (arrows). Also, partial destruction of the mandible (C) is visible (arrowheads).

Nine months later, an increase of AFP was detected for the first time, although still only slightly above the normal limit, at 13.6 U/mL. The patient clinically deteriorated. In the follow-up CT examination, a mass in the right adrenal gland was discovered (Figure 4). No additional work-up of the adrenal mass was performed. It was considered to be a metastasis because the adrenal mass was not present on previous CT exams. No additional symptoms appeared until six months later when he started to complain of paresthesia and pain of the right jaw, as well as headaches and vertigo. On a CT scan of the head and neck, a mass of 35 mm x 30 mm x 12 mm was found in the right masticatory space; lateral to the lateral pterygoid muscle, and medial to the ramus of the mandible (Figure 5A,B). The mass opacified intensely in the arterial phase. Additionally, the cortex of the mandibular ramus showed partial destruction (Figure 5C). The differential diagnosis included a metastasis, sarcoma, and osteosarcoma.

**Figure 6:**
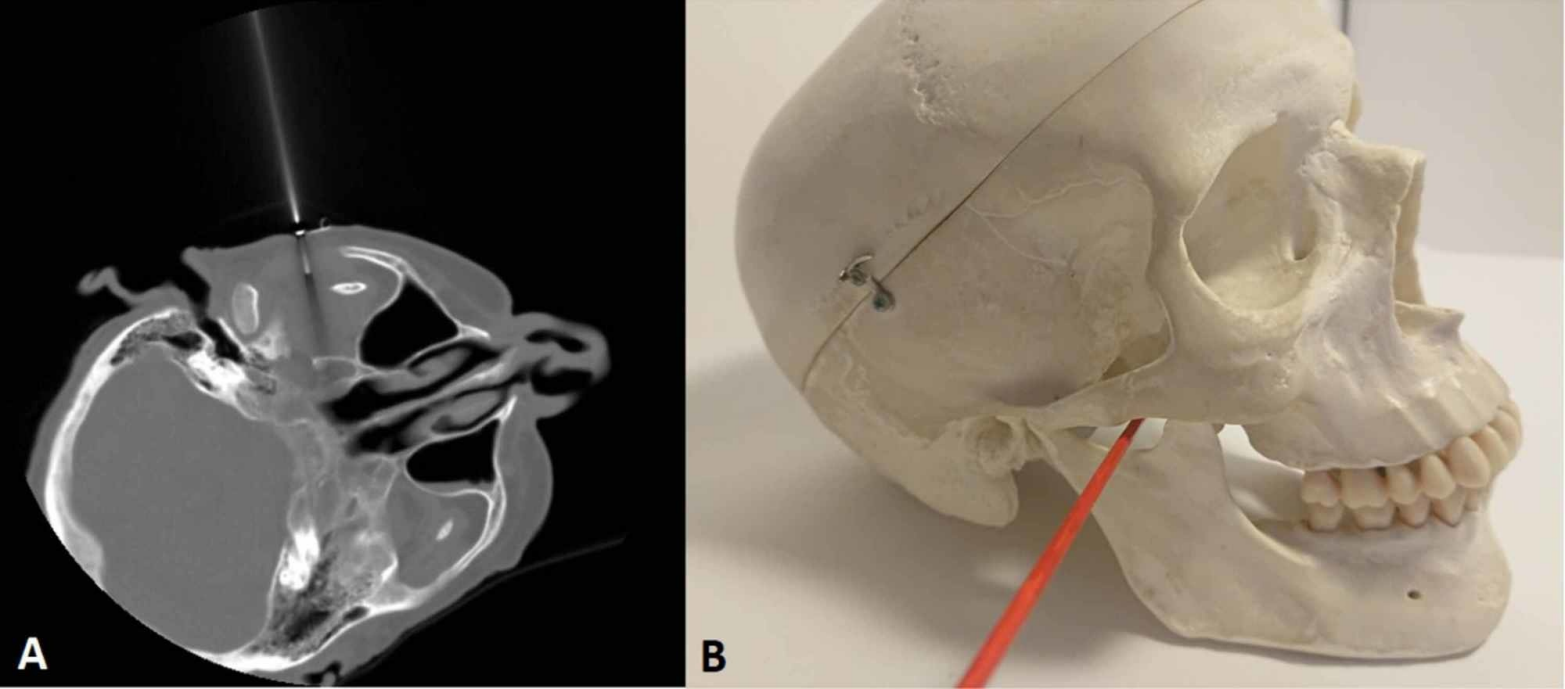
CT-guided biopsy. Under the CT guidance, a needle is inserted in the mass via the subzygomatic approach. (A) A CT confirming the correct needle insertion. (B) Schematic demonstration of the subzygomatic approach that was used.

A CT-guided percutaneous needle biopsy of the lesion was performed via a subzygomatic approach (Figure 6). A cutting type of needle was used. The histopathological examination revealed a metastatic lesion from the HCC of a trabecular type with a production of bile pigment. 

Palliative treatment was started, including radiotherapy for the lesion in the masticatory space. Two months later, the patient died after several cardiac complications and eventual multiorgan failure. This was four years after the discovery of the first liver change and two years after establishing the HCC diagnosis.

## Discussion

In the presented case the diagnosis of HCC was delayed probably owing to the atypical appearance at the first US and CT examinations. The findings were misleading toward the FNH, which does not have any potential for malignant transformation [7]. At the pre-contrast CT, the FNH is typically iso- or hypoattenuating and possibly hyperattenuating if the rest of the liver is fatty. After contrast, it usually demonstrates early arterial enhancement, which sustains in the portal phase with centrifugal (inside-out) filling. It typically has a prominent central artery and a scar with radiating fibrous septa. Due to the lack of portal veins, it has a centrifugal (spoke-wheel) flow [6, 8-9]. The main differential diagnosis of the typical FNH includes hepatic adenoma, which on contrast-enhanced imaging demonstrates similar enhancement in the arterial phase but centripetal filling (outside-in) in the portal phase. Hepatic adenoma does have a small chance of progression to HCC, especially in men [3].

Some HCC variants can also present very similarly to FNH. Early distinction is made mainly by the presence of cirrhosis or vascular invasion in HCC. Neither was present in our case. The liver was not cirrhotic, although it was steatotic. Only recent research indicates a possible risk of the progress of HCC development in the setting of non-alcoholic fatty liver disease (NAFLD) [10-11]. AFP levels are elevated in up to 80% of HCC cases [12]; in our patient, they were not increased until very late in the course of the disease. After contrast application, both HCC and FNH show early enhancement, but the wash-out is usually fast in HCC, and more gradual in FNH. In the presented case, the arterial enhancement was fast, and the portal wash-out was something in-between fast and gradual. The patient remained asymptomatic until late in the disease process. Therefore, the findings were pointing away from the diagnosis of the HCC at the initial examinations.

Unfortunately, once the diagnosis of HCC was established, surgery was no longer an option. The use of TACE and chemotherapy temporarily slowed down the disease progression. Extrahepatic metastases to the masticatory space and possibly to the adrenal gland were discovered. The metastasis in the masticatory space could have been the cause of his additional symptoms (i.e., headache, vertigo, paresthesia, and pain of the jaw). Symptomatic treatment was indicated.

The use of image-guided percutaneous needle biopsy for diagnosis of the masticatory mass is minimally invasive, which is especially valuable for patients with an already progressed disease. When dealing with nonpalpable, deep-seated head and neck lesions, CT-guided percutaneous needle biopsy is considered as a safer and more accurate option than an open surgical biopsy [13-16].

While HCC metastases to the adrenal glands are relatively common [5], metastasis to a masticatory space appears to be very rare. Due to the prolonged survival of HCC patients, there is an increase in the reported cases of extrahepatic metastases [17], which worsen an already poor prognosis.

## Conclusions

The presented case demonstrates that in liver lesions, even a typical benign radiological presentation, including Doppler, CEUS, and even CT, could be misleading. Therefore, caution is warranted with more frequent US follow-up as well as possible MRI examination, which has a higher sensitivity and specificity. With prolonged survival, the metastases may appear more often in unusual locations, such as in the masticatory space.
